# Editorial: Ligninolytic enzymes and their potential applications

**DOI:** 10.3389/fmicb.2023.1235206

**Published:** 2023-07-10

**Authors:** Abdelmageed M. Othman, Tahar Mechichi, Pankaj Chowdhary, Waleed B. Suleiman

**Affiliations:** ^1^Microbial Chemistry Department, Biotechnology Research Institute, National Research Centre, Giza, Egypt; ^2^Laboratoire de Biochimie et de Génie Enzymatique des Lipases, Ecole Nationale d'Ingénieurs de Sfax, University of Sfax, Sfax, Tunisia; ^3^Environmental Toxicology Group, CSIR-Indian Institute of Toxicology Research (IITR), Lucknow, India; ^4^Botany and Microbiology Department, Faculty of Science, Al-Azhar University, Cairo, Egypt

**Keywords:** microbial technology, bio-nanotechnology, manganese peroxidase, lignin peroxidase, laccase, ligninolytic enzymes

Versatile peroxidase, manganese peroxidase, lignin peroxidase, and laccase are ligninolytic enzymes that have lately achieved substantial economic importance in a variety of sectors including biosensors, pulp, paper, food, textile, detergent, removing endocrine disruptors, and pharmaceutical industries, cosmetics, synthetic chemistry, bioremediation, and biodegradation (Daâssi et al., 2016; Mtibaà et al., 2020; Ben Ayed et al., 2022). Ligninolytic enzymes have played an important role in biotechnological and environmental processes throughout the last few decades. Because of that, ligninolytic microorganisms have piqued the interest of researchers during screening efforts to find unique microbial producers. Ligninolytic enzymes are important players in the global carbon cycle in addition to their commercial uses. Due to the fact that they may be used in a variety of procedures, ligninolytic enzyme complexes are becoming increasingly popular for use in industry and biotechnology (Paul et al., 2023).

Researchers are looking toward enzymatic methods for developing eco-green chemistry technologies because of the drawbacks of chemical and physical methods, growing eco-friendly considerations, legal restrictions, and the increasing body of knowledge in science. Particularly since they frequently benefit the environment and have several potential uses, enzyme-assisted reactions are the focus of a lot of studies (Gad et al., 2022; Hendy et al., 2023). Recent attempts to modify enzymes may help improve the enzyme's stability and activity. As a result, efforts to enhance immobilization techniques must also be increased. Though various methods for immobilizing enzymes have been developed, many of them have the serious drawback of quickly deactivating the enzyme. Innovative immobilization techniques have been developed to create chemically bonded immobilized enzymes superior to free enzymes in terms of stability and activity (Othman et al., 2021, 2023). The ligninolytic enzymes are being researched as prospective environmental enzymes to exchange the conventional mechanical and chemical courses in several industries, comprising nanoparticle-based bio-devices, pharmaceuticals, pulp and paper, and textile industries (Singh et al., 2023). In light of this background, this particular Research Topic has been created to concentrate on innovative, cutting-edge, and promising advancements in the study of microbial ligninolytic enzymes. Twenty-three authors have contributed articles to this Research Topic. In order to meet the demands of the readers, this Research Topic contains four peer-reviewed original research articles, all of which address many aspects of this subject.

Considering that biodelignification is typically sluggish and hard to manage, it is commonly viewed as a low-efficiency process. Li et al. examined the ability of *Pleurotus ostreatus* to delignify cotton stalks present in the growth medium to increase its effectiveness and comprehend its process. The outcomes showed that all the *P. ostreatus* strains were capable of preferential lignin degradation in cotton stalks. *P. ostreatus* generated two liginiolytic enzymes, namely manganese-dependent peroxidase and laccase, with distinguished enzymatic activity levels of 62.39 and 70.17 U/ml, correspondingly; however, no lignin peroxidase could be found. It destroyed lignin by up to 54.04%, largely during the development of its mycelium. Guaiacyl (G) lignin units had a lower G/S ratio than syringyl (S) lignin units, which were more degraded, according to spectroscopic investigations showing major changes in lignin structure. These discoveries will increase the effectiveness of biodelignification and deepen our comprehension of its process.

Microorganisms that break down wood are a rich source of newly discovered enzymes. Different decomposing wood samples were examined by Ali et al., who also found a promising γ-proteobacterial strain known as *Serratia proteamaculans* AORB19 that naturally released a considerable quantity of laccase enzyme. Both qualitative and quantitative tests were performed to validate the laccase activity of strain AORB19 in the culture medium. Using the one-factor-at-a-time (OFAT) technique, significant cultural factors for laccase synthesis in submerged settings were found. The study's findings point to the strain's and its enzymes' intriguing potential for valorizing lignocellulosic wastes.

The majority of existing techniques for evaluating lytic polysaccharide monooxygenase (LPMO) activity rely on measuring H_2_O_2_, a byproduct of LPMO; however, due to H_2_O_2_'s poor thermostability, most techniques cannot measure the LPMO function in thermophilic fungi. *Thermoascus aurantiacus*, a thermophilic fungus, was measured for its LPMO activity by Yu et al. using a high-performance liquid chromatography-refractive index detector (HPLC-RID) approach. They examined the oxidation performance of *Pichia pastoris*-expressed recombinant and three of its mutants. They came to the conclusion that the technique offers an efficient activity test method for LPMOs of thermophilic fungi.

Aflatoxins are naturally occurring, highly poisonous secondary metabolites that seriously endanger human health, pollute the environment across the world, and squander food and feed resources. Aflatoxin detoxification techniques and technology are desperately needed in the long run. The development of three recombinant *Kluyveromyces lactis* strains, followed by the production of the three manganese peroxidases in these bacteria and the degradation of aflatoxin B1 (AFB1) utilizing the Fermentation supernatants, were all described by Xia et al.. After two treatments, the recombinant strain GG799 (pKLAC1-Phcmnp) supernatants destroyed more than 90% of the AFB1 in the peanut samples. The findings declare the possibility of using the food-grade recombinant yeast strain and its enzyme in the feed or food sectors.

In conclusion, ligninolytic enzymes are now more economically valuable across a range of businesses. Due to their versatility in a range of processes, ligninolytic enzyme complexes are being used increasingly often in biotechnology and industry. This Research Topic is an effort to focus on new, cutting-edge, and intriguing advances in our understanding of microbial ligninolytic enzymes. Future economic and environmental considerations should take into account the low cost of ligninolytic enzyme synthesis using trash found all around us to help address various environmental challenges. In order to increase the potential for enzyme immobilization, studies about using new functional immobilization supports with various functional groups should be taken into consideration. Using green and environmentally friendly alternatives can be a promising area for further research and new potential applications.

## Author contributions

All authors listed have made a substantial, direct, and intellectual contribution to the work and approved it for publication.
